# The Effects of Azithromycin Modified Triple Antibiotic Paste in Resolving Periapical Inflammation

**DOI:** 10.1016/j.identj.2025.100895

**Published:** 2025-07-15

**Authors:** Tingting Meng, Zhengyang Wang, Haocong Li, Zhaoguang Ouyang, Chenxi Yang, Tengye Lian, Yao Zhang, Huixu Li, Pingping Bao, Dayong Liu

**Affiliations:** a Department of Endodontics, Tianjin Stomatological Hospital, School of Medicine, Nankai University, Tianjin, 300041, China; b Tianjin Key Laboratory of Oral and Maxillofacial Function Reconstruction, Tianjin, 300041, China; c Tianjin Key Laboratory of Oral Soft and Hard Tissues Restoration and Regeneration, Tianjin Medical University School of stomatology, Tianjin Medical University, Heping, Tianjin, China; d Shanghai Engineering Research Center of Tooth Restoration and Regeneration & Tongji Research Institute of Stomatology & Department of Endodontics, Shanghai Tongji Stomatological Hospital and Dental School, Tongji University, Shanghai, China; e Department of Pharmacology, State Key Laboratory of Experimental Hematology, Tianjin Key Laboratory of Inflammatory Biology, The Province and Ministry Co-Sponsored Collaborative Innovation Centre for Medical Epigenetics, NHC Key Laboratory of Hormones and Development, Chu Hsien-I Memorial Hospital and Tianjin Institute of Endocrinology, School of Basic Medical Sciences, Tianjin Medical University, Tianjin, China

**Keywords:** Azithromycin, triple antibiotic paste (TAP), Modified triple antibiotic paste (mTAP), Periapical inflammation, Immunomodulation, Anti-inflammatory

## Abstract

**Objective:**

This study aimed to assess the anti-inflammatory and antibacterial properties of traditional and modified triple antibiotic paste. The 16S rDNA was employed to analyse the root canal microbial community's characteristics pre- and post-treatment.

**Methods:**

Rabbits with periapical inflammation (AP) were treated with both pastes. Micro-CT was performed to compare the periapical lesion area, followed by HE staining. Inflammatory factors and microorganism were collected for analysis. T-tests and ANOVA were used to analyse the effects of the 2 pastes on tooth discoloration.

**Results:**

Compared with the control group, apical lesions and the expression of inflammatory cytokines was decreased, along with a reduction in bone destruction. Antibacterial experiments showed that the 2 antibiotics had similar effects on the common pathogens of AP. Compared with the TAP, mTAP does not cause discoloration of teeth. 16Sr DNA analysis revealed variations in periapical bacteria composition. The contents of common pathogens of AP in TAP and mTAP groups were lower than those in control group.

**Conclusions:**

Azithromycin has the potential to serve as an alternative to minocycline. The 16S rDNA data highlights the intricate nature of the root canal microbiome, promising deeper insights into the correlation between microbes and inflammation.

## Introduction

Periapical inflammation (AP) is a chronic inflammatory condition resulting from root canal infection, with its progression influenced by the host's immune and inflammatory responses. Root canal therapy (RCT) is the conventional approach for treating fully developed permanent tooth root canal disease, while apexification or apexogenesis is the customary procedure for managing immature permanent teeth with pulp lesions.^1^, ^2^, ^3^

Although, infection control plays an important role in these treatments, often overlooked, the impact of inflammatory responses on tissue regeneration can result in failures in cases of young permanent tooth regeneration or revascularisation. The objective of revascularisation is not only to promote healing of periapical lesions and resolution of signs and symptoms but also to facilitate continued root development and strengthen dentin tissue to prevent potential root fracture.^4^^,^^5^ In clinical practice, triple antibiotic paste (TAP), which containing metronidazole, minocycline, and ciprofloxacin, is often used for root canal disinfection. The efficacy of TAP in revascularisation has been acknowledged. However, antibiotic pastes, besides side effects such as the risk of tooth discoloration,^6^ lack anti-inflammatory components.

Azithromycin (AZM), a second-generation macrolide antibiotic with a broad antibacterial spectrum, possesses not only antibacterial properties similar to erythromycin and clarithromycin but also anti-inflammatory and immunomodulatory properties.^7^^,^^8^ It has been shown to shift macrophage activation towards the M2 phenotype,^9^ thereby inhibiting the synthesis of proinflammatory cytokines (such as IL-1, IL-8, and TNF-α), neutrophil accumulation, and superoxide anion production by neutrophils.^10^, ^11^, ^12^ Furthermore, AZM regulates macrophage activation from proinflammatory M1 macrophages to proresolving M2 macrophages, promoting efferocytosis and wound healing.^11^^,^^13^^,^^14^ Previous studies have demonstrated that AZM can inhibit TNF-α-induced apoptosis of human periodontal ligament stem cells (PDLSCs) and promote osteogenesis differentiation.^15^ The concentration of AZM in gingival crevicular fluid is significantly higher than in serum, making it effective in the treatment of periodontitis.^16^ The adjunctive role of AZM in periodontitis treatment has been validated in multiple studies.^17^ Additionally, AZM significantly reduces the destruction of alveolar bone in experimental periodontitis in rats and improves the bone trabecular structure.^18^ AZM and ampicillin (AMP) can attenuate experimental periapical periodontitis lesions and reduce periapical bone loss in C57BL/6J mice .^19^" However, AZM is rarely used on young permanent teeth and its effect of treating chronic periapical periodontitis remains unclear.

The objective of this study was to substitute minocycline with AZM to formulate mTAP and investigate its effects on pulp regeneration/revascularisation of young permanent teeth by controlling inflammation, aiming to provide insights for tissue regeneration under inflammatory microenvironment.

In this study, we established an rabbit experimental periapical periodontitis model and performed antibacterial assays, discoloration experiments, inflammatory cytokines analyses, hematoxylin and eosin (HE) staining, micro-computed tomography (Micro-CT) imaging, and 16S rDNA sequencing to analyse the microbiome composition in the infected root canals of experimental rabbits and to observe the effects of mTAP and TAP on root canal microorganisms.

## Materials and methods

### Animal experiment

The TAP comprised ciprofloxacin, minocycline, and metronidazole, whereas the mTAP consisted of metronidazole, AZM, and ciprofloxacin. Each medication's components were mixed in a 1:1:1 ratio, and both TAP and mTAP were dissolved in sterile water to achieve a concentration of 0.1 mg/mL, consistent with pharmacologically active antibiotic concentrations in plasma. In animal experiments, both medications were mixed with sterile water to attain a concentration of 1 mg/mL, resulting in a runny paste consistency.

Rabbits aged 3 months and 2 kg were selected for the experiment, with the first maxillary premolars accessed under general anesthesia. Following pulp removal, the teeth were left open for 3 weeks. One side served as the control group, receiving distilled water, while the other side constituted the experimental group, where TAP or mTAP was applied. After 2 weeks, the involved teeth were disinfected using a sterile cotton pellet soaked in sodium hypochlorite. Samples were collected by repeatedly touching the canal walls with a loose file (typically a size 25 K-file) in both the control and experimental groups, without actual filing. After several repetitions, the K-file was placed into an EP tube containing cell preservation solution, and sterile paper points were inserted into the root canal with tweezers for approximately 30 seconds. The samples were then stored at -80°C, and 16S rDNA analysis was performed to detect bacterial composition in different groups. Similarly, the K-file and paper points were placed as close to the apex as possible using the same method. After 30 seconds, they were removed and stored in an EP tube containing 100μl PBS at -80°C for enzyme-linked immunosorbent assay (ELISA) to detect inflammatory factor levels in different groups. The animal use protocol listed has been reviewed and approved by the Animal Ethical and Welfare Committee (AEWC) (No. IRM-DWLL-2022259).

### Antibacterial experiment in vitro

Three common anaerobic pathogenic bacteria found in periapical inflammatory root canals were selected for study: *Enterococcus faecalis* (E. faecalis), *Fusobacterium nucleatum* (F. nucleatum), and *Streptococcus mutans* (S. mutans). Pure cultures of these bacteria were prepared and inoculated into Brain Heart Infusion (BHI) liquid medium until reaching the logarithmic growth phase. The bacterial solution was then diluted tenfold and added to 96-well plates along with culture medium, TAP and mTAP, respectively. Cultures were maintained for 10 hours under aseptic constant temperature conditions, with optical density (OD) values measured and recorded every 2 hours.

Following the assessment of mTAP's impact on suspended bacterial growth, its effect on bacterial biofilm formation was also investigated. Bacteria were cultured to form biofilms in a suitable culture medium. The control group received only the culture medium, while the other 2 groups were exposed to TAP and mTAP, respectively. After 24 hours of incubation, the biofilms were washed twice with phosphate-buffered saline (PBS), stained with live/dead bacterial dyes, and incubated at 37°C in the dark. Subsequently, the biofilms were examined under a confocal microscope.

### Tooth discoloration

Nine extracted human mandibular premolars were utilised in this study. Following cleaning, all teeth underwent radiographic examination to confirm the absence of resorption or prior endodontic treatment. The apices of all teeth were intentionally opened to approximately 1.5 mm to mimic the condition of young permanent teeth. After sterilization in an autoclave, the teeth were immersed in saline for 72 hours. Subsequently, access to the pulp chamber was achieved, and the entire pulp contents along with the coronal portion of the root canal were removed using a size 15 K-file and saline solution.

The samples were then divided into 3 groups: the control group received no medication, while the other 2 groups were treated with TAP and mTAP, respectively. A cotton pellet was inserted into the pulp chamber, and the access opening was sealed with Cavit. The samples were stored at 37°C for 3 weeks, during which photos were taken weekly to record the Lab* values. The L* value represents lightness, ranging from 0 for black to 100 for white; a* indicates the amount of red (positive values) or green (negative values); and b* signifies the amount of yellow (positive values) or blue (negative values). The constant difference in color (ΔE) was calculated using the formula: ΔE = [(ΔL)2 + (Δa)2 + (Δb)2]1/2.

## Results

### Anti-inflammatory effect of modified TAP

**Fig. 1:**
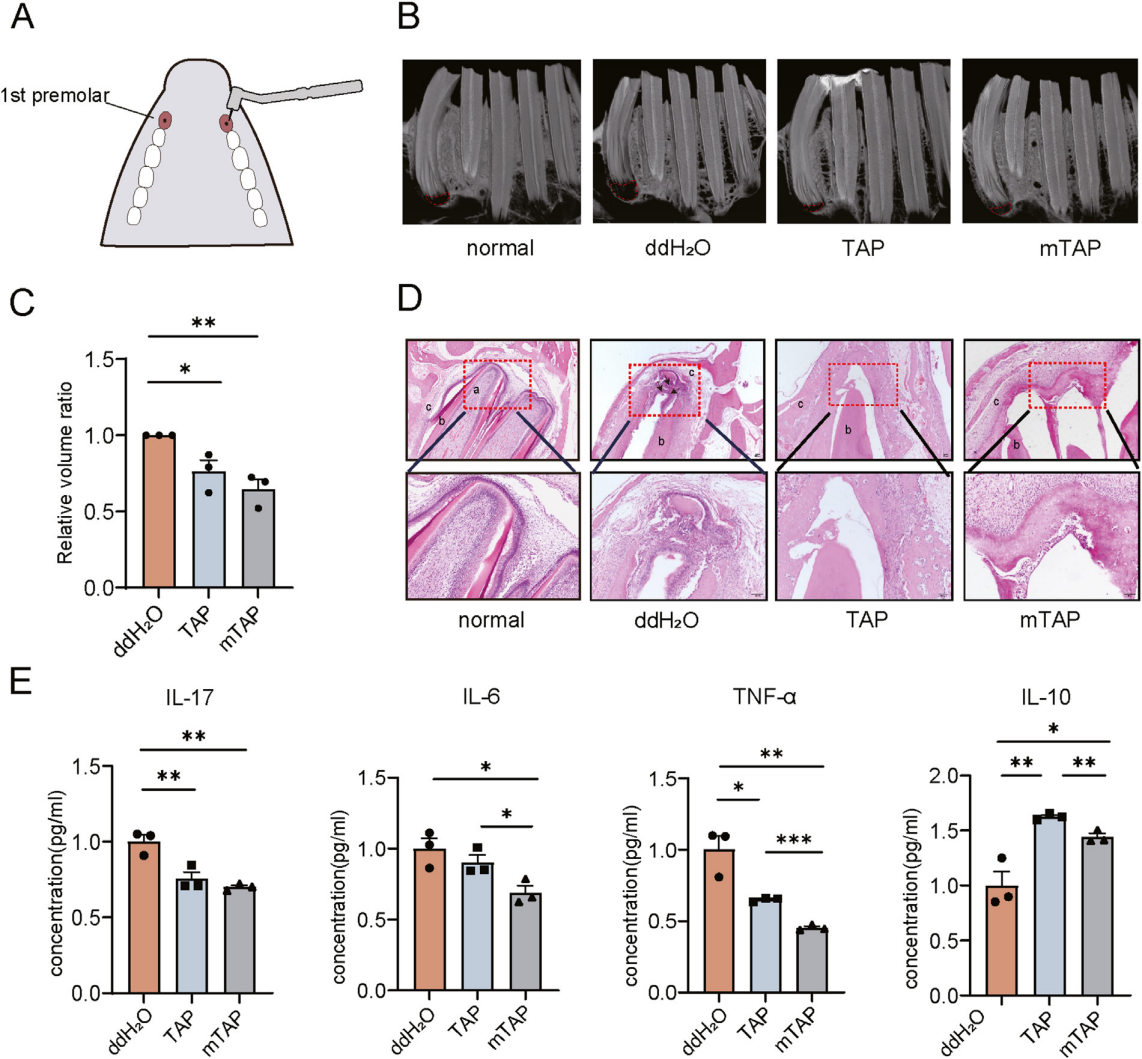
The anti-inflammatory and immunomodulatory effects of mTAP are similar to those of TAP. (A) Bilateral maxillary first premolars of rabbits were pulp-removed and opened for 21 days. Antibiotics were added to one side, and the other side was temporarily sealed without treatment. (B) Representative micro-CT images of the control, TAP and mTAP groups after 14 days of treatment. Both antibiotic treatment groups showed a reduction in periapical lesion volume compared with the control group. (C) The average lesion size of the control group was used as the baseline to calculate the lesion volume ratio of the experimental group to the control group (n = 3). (D) Hematoxylin-eosin staining showed that compared with the control group, the degree of periapical inflammatory infiltration and bone destruction were reduced in the experimental group. Both TAP and mTAP considerably resolved inflammation. Original magnification: × 40. (E) Enzyme-linked immunosorbent assay showed that both antibiotics had immunomodulatory ability in vitro, and mTAP had a stronger effect. This may be due to the fact that AZM can shift macrophage activation to the M2 phenotype. All values are expressed as means ± SEM. *p < .05, **p < .01, ***p < .001.

Following anesthesia administration to rabbits, a model of AP was established in the maxillary first premolars (Figure 1A). After 21 days, either TAP or mTAP was applied and sealed on one side, while the opposite side applied distilled water. Subsequently, after 14 days of treatment with TAP or mTAP, micro CT imaging was utilised to assess the effect of antibiotic therapy on the original periapical lesions (Figure 1B). Using software, the volume of the lesion surrounding the periapical region was calculated and compared. The mean lesion size of the control group served as the baseline, and the ratio of lesion volume between the experimental group and the control group was calculated. Compared to baseline, the size of periapical lesions in rabbits treated with both antibiotics decreased significantly at day 14, with mTAP treatment resulting in a significantly greater reduction in periapical lesion size compared to TAP (Figure 1C).

Histological examination of the samples was conducted to confirm the effect of the 2 antibiotics on AP. In the control group, a large number of inflammatory cells infiltrated, and alveolar bone damage was severe. In contrast, in the TAP and mTAP groups, bone destruction was reduced, and there was a smaller amount of inflammatory infiltration (Figure 1D). To elucidate possible mechanisms for the differences in periapical wound healing and immunomodulatory activity, the effects of the 2 antibiotics on inflammatory response were examined in vitro (Figure 1E). The results demonstrated that mTAP exhibited a more potent immunomodulatory effect in vitro compared to TAP.

### The destructive and inhibitory effects of TAP and mTAP on common root canal bacteria were assessed in vitro

**Fig. 2:**
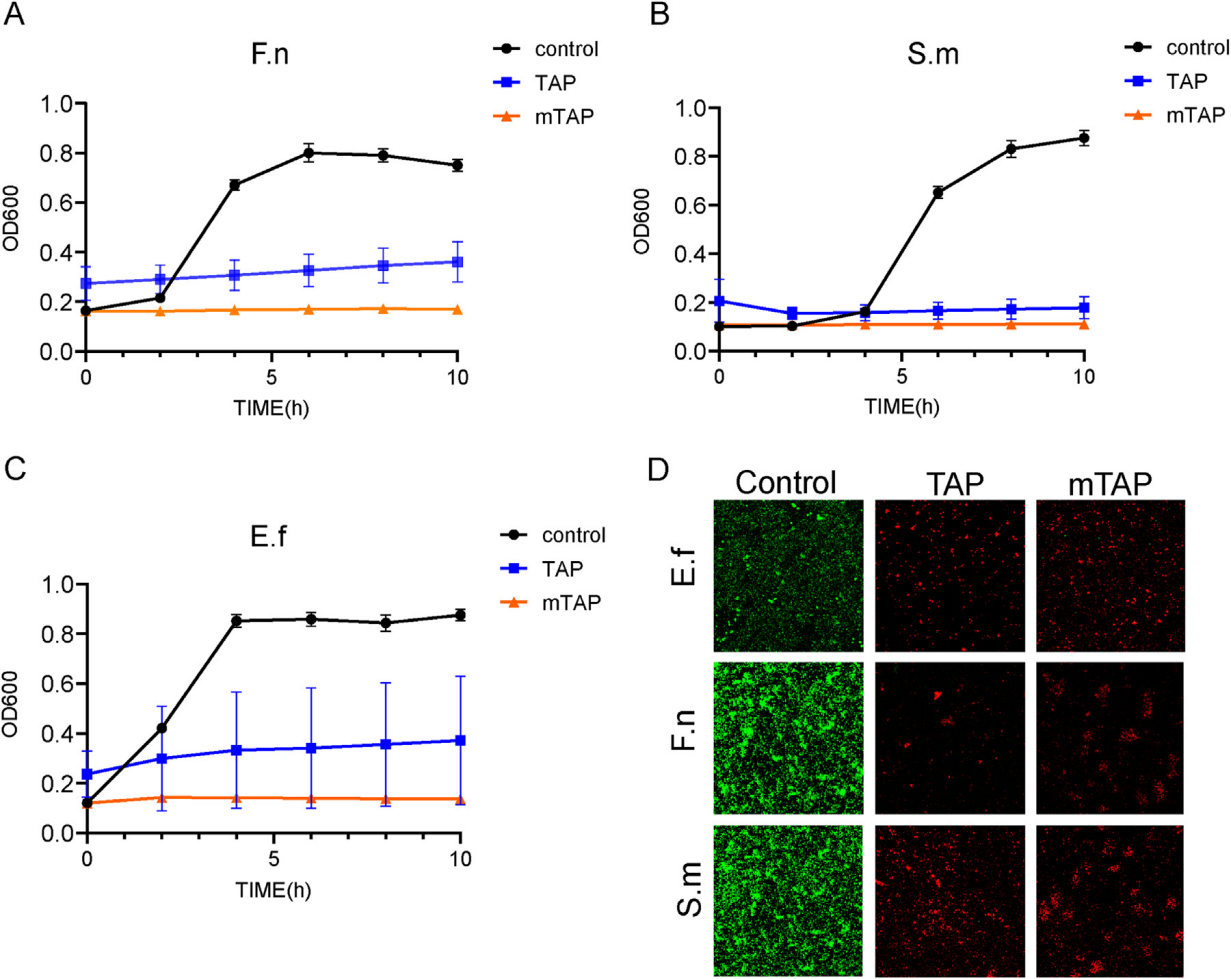
The antimicrobial action of 2 antibiotics in vitro. (A-C) Effects of 2 antibiotics on suspensions of common root canal pathogenic microorganisms. (D) Live and dead bacteria staining was used to observe the destruction of bacterial biofilms by the 2 antibiotics, green represents live bacteria and red represents bacteria that have been destroyed.

The common pathogenic bacteria found in the root canal during AP were *Enterococcus faecalis* (*E. faecalis*), *Fusobacterium nucleatum* (*F. nucleatum*), and *Streptococcus mutans* (*S. mutans*), all of which are anaerobic bacteria. These bacteria were cultured to the logarithmic stage and then subjected to separate treatments. In the experimental group, either 0.1mg/ml TAP or mTAP was added, while the control group received only bacterial solution. The cultures were then incubated at 37°C for 10 hours in an anaerobic environment, with absorbance values at 600nm measured every 2 hours (Figures 2A–C).

Following the observation of the potent inhibitory effects of both antibiotics on the suspended bacterial solution, subsequent biofilm formation was induced. Similarly, both TAP and mTAP demonstrated remarkable efficacy in eradicating bacterial biofilms (Fig 2.D). In vitro experiments conducted with root canal microorganisms revealed that TAP and mTAP exhibited comparable destructive effects.

### mTAP significantly reduced the risk of crown discoloration compared to TAP in vitro

**Fig. 3:**
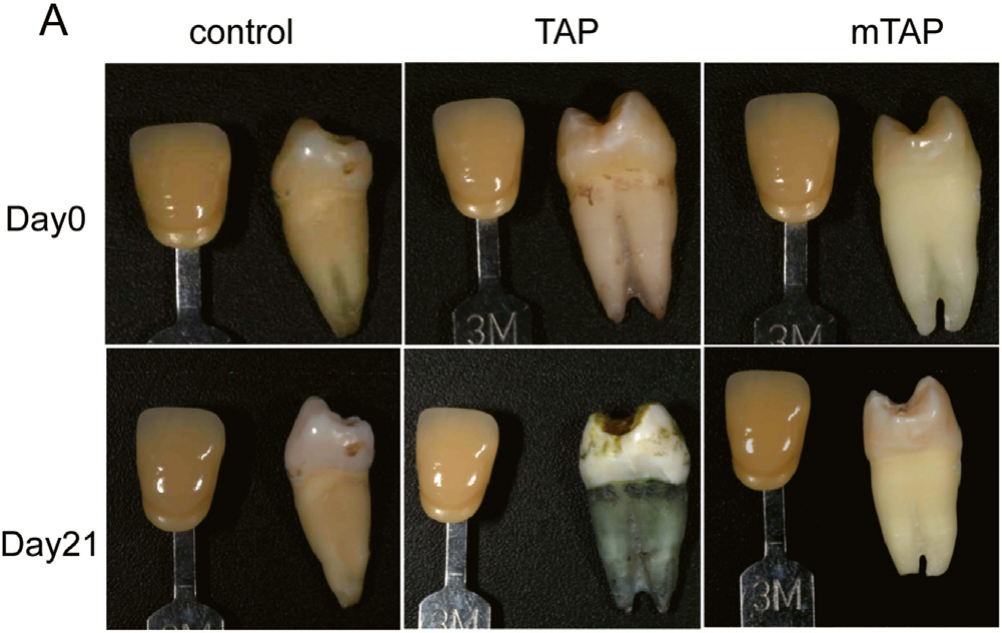
The effect of 2 antibiotics on tooth colour. (A) The teeth without caries or pulp disease were selected, and the periapical opening was 1.5mm to simulate young permanent teeth. Antibiotics were added in the experimental group, no treatment was done in the control group. Cotton balls were placed in the medullary cavity and prepared by blocking the channel with Cavit. Samples were stored at 37°C for 3 weeks and photographed weekly to observe colour changes and record L*a*b* values. (n = 3). (B) Changes in ΔE of the 3 groups over 3 weeks. L* indicates lightness; a* means the amount of red or green; b* means the amount of yellow or blue. ΔE = [(ΔL)2 + (Δa)2 + (Δb)2]1/2.

The TAP has been associated with tooth discoloration issues due to the presence of minocycline, a drug from the tetracycline family. In this study, we investigated whether similar discoloration occurs when minocycline is replaced with AZM. Following access opening, both TAP and mTAP were sealed in the pulp chamber for a duration of 3 weeks. The teeth were photographed weekly to record any changes in color (Figure 3A).

A significant difference (ΔE) in tooth color was observed in all teeth treated with TAP compared to control teeth. However, in comparison to the control group, the change in color observed in the mTAP group was not significant (Figure 3B).

### 16S rDNA sequencing

**Fig. 4:**
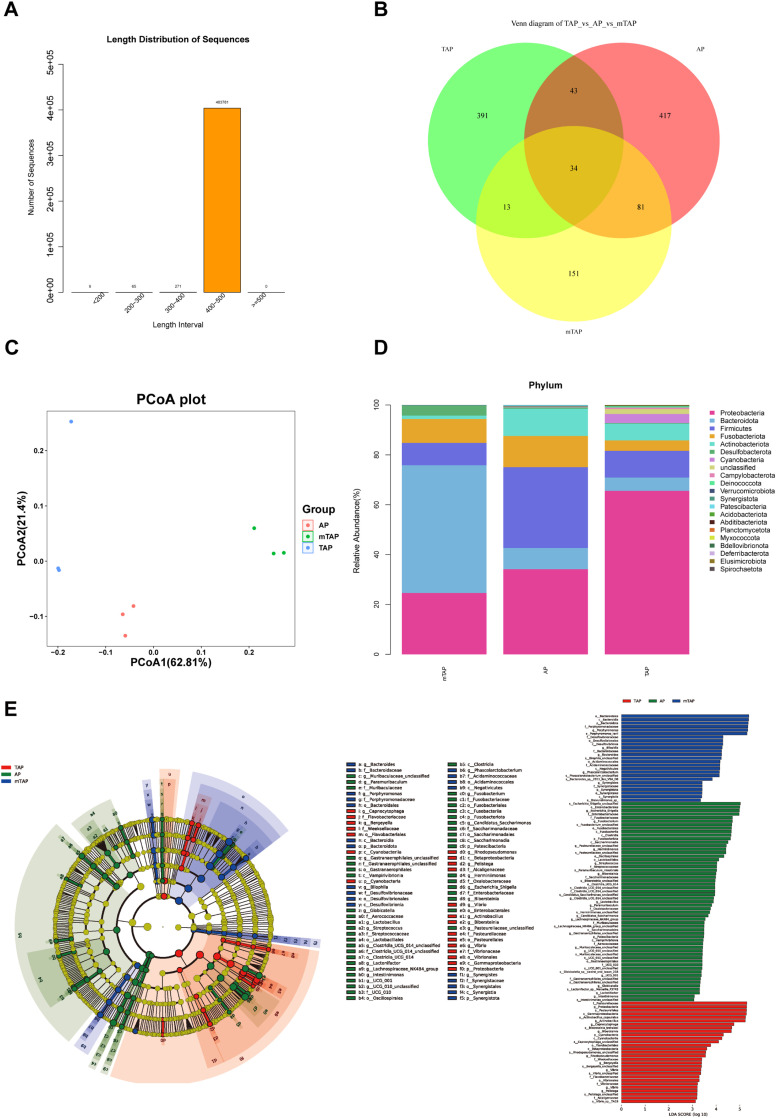
16Sr DNA sequencing was used to detect bacterial genera in the root canal specimens of rabbits with periapical periodontitis. (A) The length distribution of sequence. (B) VENN diagram show the number of ASVs common or unique to the 3 groups of bacterial communities. (C) PCoA (Principal Coordinates Analysis) When the biological replicate samples are closer together and the distance between different groups is longer, the treatment is effective or the difference is significant. The percentage of the horizontal and vertical coordinates indicates the degree to which the first and second axes explain the sample differences. (D) Relative abundance of the 3 groups of samples at the “phylum” level. (E) LDA effect size (LEfSe) was used for multi-group analysis to find species with significant differences(biomarker) in abundance between different groups. Species with significant differences are indicated directly in the figure.

All nine analysed samples tested positive for the presence of bacterial DNA. Following quality control measures, a total of 616,457 16S rDNA gene sequences were detected (Figure 4A). The number of common and unique Amplicon Sequence Variants (ASVs) in each group can be visually represented by a Venn diagram (Figure 4B).

In the principal coordinates analysis plot (Figure 4C), different colors represent different groups, and samples closer together indicate a more similar microbial composition and structure, with smaller differences observed.

For classification analysis, we selected the 30 species with the highest abundance. At the phylum level, the highest relative abundance was observed for “*Proteobacteria*” and “*Firmicutes*,” followed by “*Fusobacteriota*” and “*Actinobacteriota*” in the AP group. Conversely, in the mTAP group, the relative abundance of bacterial species decreased overall, including common species found in periapical lesions. However, the relative abundance of “*Bacteroidota*” increased in the mTAP group, and “*Proteobacteria*” increased in the TAP group (Figure 4D).

LDA Effect Size analysis was utilised to identify species with significant differences in abundance between groups. Circles in the plot represent taxonomic levels, with larger nodes indicating higher abundance of that species (Figure 4E). The results highlighted major differential species at the phylum level, including “*Proteobacteria*”, “*Firmicutes*”, “*Fusobacteriota*”, and “*Actinobacteriota*” in the AP group; “*Bacteroidota*” in the mTAP group; and “Proteobacteria” in the TAP group, consistent with previous findings.

## Discussion

Treatment of immature permanent teeth with necrotic pulp or AP typically involves apexification procedures using calcium hydroxide to induce periapical hard tissue barrier formation.^20^, ^21^, ^22^ However, a drawback of traditional apexification techniques is the potential weakening of root strength due to the short- or long-term use of calcium hydroxide.^23^^,^^24^ An alternative approach involves creating artificial barriers using materials such as MTA, which reduces the number of appointments and treatment time.^25^^,^^26^ However, neither method typically results in further root development. The term “revascularisation” was first introduced by Iwaya et al.,^27^ for the development of teeth and regeneration of the pulp-dentin complex, utilising TAP for disinfection.

Although the role of TAP in revascularisation is well recognised, it can lead to tooth discoloration, and challenges in its removal from the root canal due to its paste form. Tooth discoloration during regenerative endodontic treatments is primarily attributed to the contact between minocycline in TAP and the dentin wall.^27^^,^^28^ To mitigate the risk, we innovatively substituted minocycline with AZM, which not only exhibits antibacterial effects but also possesses anti-inflammatory and immunomodulatory properties.^11^^,^^29^ In this study, after using mTAP, a reduction in the volume of periapical lesion was radiographically observed. Histological examination revealed decreased inflammatory cell infiltration and bone destruction, indicating a resolution phase of infection-induced AP. Furthermore, levels of proinflammatory factors (e.g., IL-6, IL-17, TNF-α) decreased, while anti-inflammatory factors (e.g., IL-10) increased, consistent with previous studies^11^ demonstrating the immunomodulatory effects of macrolides.

Bacteria and their metabolites are widely recognised as the primary etiological agents of pulp necrosis and periapical lesions. Consequently, the complete eradication of bacteria from the root canal system during endodontic treatment is considered a critical step for successful therapeutic outcomes.^30^^,^^31^ In our in vitro experiments, we selected 3 prevalent pathogenic microorganisms commonly associated with root canal infections, *Streptococcus mutans,* a Gram-positive coccus, is frequently identified as a common pathogen in root canal infections.^32^ *Enterococcus faecalis* is a resilient organism that plays a pivotal role in the pathogenesis of persistent periapical lesions following RCT. It is frequently detected in a substantial proportion of failed root canal treatments and has the ability to survive within the root canal either as a single species or as a dominant member of a bacterial consortium.^33^^,^^34^ *Fusobacterium nucleatum* is a Gram-negative anaerobic bacterium with significant pathogenicity, implicated as a key etiological agent in both periodontitis and pulpitis.^35^ *F. nucleatum* often dominates the microbial flora in infected root canals and, at times, exhibits the highest detection rate among the bacterial species present.

Previous studies have indicated that cells cannot survive contact with dentin treated with high concentrations of TAP.^36^ However, concentrations of 0.1–1 mg/mL are reported to have no harmful effects and are sufficient to eradicate bacteria in the root canal.^37^ In this study, both TAP and mTAP were used at a concentration of 1mg/ml, which showed no adverse effects on stem cells. Considering the global concern over antibiotic resistance, adjuvant antibiotic therapy should be restricted to cases with systemic involvement or in immunocompromised/susceptible patients.

The transition of drugs from the laboratory to clinical application is a lengthy and intricate process, with animal testing serving as a critically important intermediary step. In this experiment, rabbits were selected as the animal model due to their economic feasibility, high survival rates, and ease of handling. Furthermore, the apical region of rabbit teeth exhibits an open structure, resembling that of young permanent teeth. We successfully established an experimental rabbit model of first maxillary premolar AP. In vitro antibacterial experiments involved 3 common oral pathogenic microorganisms, demonstrating the efficacy of both drugs. Animal experiments were conducted to further verify the antibacterial properties of AZM. Next Generation Sequencing has enhanced our understanding of microbiota composition in various physiological and pathological conditions. Using 16S rDNA analysis, we investigated the microbiome composition of samples obtained from rabbit root canals with AP. In the AP group, the relative abundance of common AP bacteria such as *Proteobacteria, Firmicutes, Fusobacteriota*, and *Actinobacteriota* was high, consistent with previous literature. However, after antibiotic use, the relative abundance of certain bacterial species actually increased. In the TAP group, *Proteobacteria* increased, while in the mTAP group, *Bacteroidota* increased. This phenomenon may be attributed to several factors. 16S rDNA sequencing-based microbiome studies have inherent limitations, resulting in relatively quantitative rather than absolute results. Additionally, outcomes may be influenced by biases arising from differences in PCR amplification, sequencing efficiency, and reference databases employed for sequence analysis. Moreover, it is important to note that only approximately 15% of bacterial genomes contain single copies of the 16S rDNA gene, while most phyla may harbor species with one or more copies.^38^

Another limitation of our study is the relatively small sample size. Including additional samples from animal experiments would likely enhance the efficacy of further investigations into the impact of AZM on bacterial microorganisms.

## Conclusion

Leveraging the anti-inflammatory and immunomodulatory attributes of AZM, the mTAP exhibits potent antibacterial and anti-inflammatory properties without inducing tooth discoloration. This substitution presents a novel treatment approach for clinical pulp revascularisation, potentially supplanting the conventional TAP.

## Conflict of interest

The authors declare no potential conflicts of interest.
